# Feather Pecking in Non-Beak-Trimmed and Beak-Trimmed Laying Hens on Commercial Farms with Aviaries

**DOI:** 10.3390/ani11113085

**Published:** 2021-10-28

**Authors:** Angela Schwarzer, Christina Plattner, Shana Bergmann, Elke Rauch, Michael Erhard, Sven Reese, Helen Louton

**Affiliations:** 1 Chair of Animal Welfare, Ethology, Animal Hygiene and Animal Husbandry, Department of Veterinary Sciences, Faculty of Veterinary Medicine, LMU Munich, 80539 Munich, Germany; plattner@cso.net (C.P.); S.Bergmann@lmu.de (S.B.); rauch@lmu.de (E.R.); michael.erhard@lmu.de (M.E.); 2 Chair of Anatomy, Histology and Embryology, Department of Veterinary Sciences, Faculty of Veterinary Medicine, LMU Munich, 80539 Munich, Germany; sven.reese@lmu.de; 3 Animal Health and Animal Welfare, Faculty of Agricultural and Environmental Sciences, University of Rostock, 18059 Rostock, Germany; helen.louton@uni-rostock.de

**Keywords:** layer, severe feather pecking, animal welfare, animal behavior, poultry

## Abstract

**Simple Summary:**

Severe feather pecking (SFP) is a major animal welfare problem in layers. It results in pain and injuries in the affected animal. SFP is a behavioral disorder and should not be confused with aggressive pecking. The aim of our study was to observe the pecking behavior of layers on farms with flock sizes common in practice and to identify possible influencing factors. We found that SFP occurred in all flocks, but the pecking rate varied widely between flocks. A low stocking density and the provision of a winter garden or free range (or both) had a positive effect and reduced SFP. Keeping mixed flocks of brown and white layers was a risk factor for SFP. SFP occurred mainly in the litter area and only rarely on perches. This finding emphasizes the importance of providing enough litter, litter areas and environmental enrichment. Aggressive pecking and SFP were correlated, which may indicate a higher stress level in the flock. Beak trimming reduced pecking rates but did not entirely prevent SFP. Instead of subjecting chicks to this potentially painful procedure, reasons for SFP should be addressed. SFP remains a multifactorial problem, but in recent years, many risk factors have been identified and included in best-practice recommendations, allowing the housing of non-beak-trimmed layers.

**Abstract:**

Severe feather pecking (SFP) is a major animal welfare problem in layers. It results in pain and injuries in the affected animal. It was the aim of this study to gain insight into the actual pecking behavior of laying hens kept on commercial farms with flock sizes common in practice. We observed aggressive pecking and SFP in non-beak-trimmed and beak-trimmed flocks of laying hens and investigated possible influencing factors. The study took place on eight conventional farms in Germany with aviaries, including three farms with a free range and a winter garden, one with a free range and one with a winter garden. Pecking behavior was observed during three observational periods (OPs): OP 1, at the peak of the laying period between the 28th and 33rd week of life; OP 2, in the middle of the laying period between the 42nd and 48th week of life; and OP 3, at the end of the laying period between the 63rd and 68th week of life in one laying period. Videos were analyzed using behavior sampling and continuous recording. We found that SFP occurred in all flocks, but the pecking rate differed significantly between the flocks. SFP correlated positively with the number of hens per square meter of usable area, with statistical significance in the litter area (*r* = 0.564; *p* = 0.045). The multivariate analysis revealed that access to a winter garden or free range significantly reduced the SFP rate on perches (*p* = 0.001). The stocking density (number of birds per usable square meter) had a significant influence on the SPF rate in the nest-box area (*p* = 0.001). The hybrid line had a significant effect on the SFP rate on perches and in the nest-box area (*p* = 0.001 each). Lohmann Brown hens in mixed flocks had a higher SFP rate (significant in OP 2) than those in homogeneous flocks, indicating that mixed flocks may be a risk factor for SFP. Lohmann Brown hens pecked significantly less than Dekalb White hens in the litter area (*p* = 0.010) and in the nest-box area (*p* = 0.025) and less than Lohmann Selected Leghorn hens in the litter area (*p* = 0.010). Lohmann Brown and Lohmann Selected Leghorn hens showed increasing SFP rates during the laying period. All hybrid lines had significantly higher SFP rates in the litter area, followed by the nest-box area and perches. These findings emphasize the importance of providing enough litter, litter areas and environmental enrichment. We found a significant positive correlation between aggressive pecking and SFP—in OP 1: rho (Spearman) = 0.580, *p* < 0.001; OP 2: rho = 0.486, *p* = 0.002; and OP 3: rho = 0.482, *p* = 0.002 (*n* = 39) —indicating that SFP may lead to a higher stress level in the flock. Beak trimming reduced pecking rates but did not entirely prevent SFP. Instead of subjecting chicks to this potentially painful procedure, reasons for SFP should be addressed. In conclusion, our data suggest a positive influence of a lower stocking density and the provision of a winter garden or free range for additional space. The hybrid line had a significant influence on the feather-pecking rate on perches and the nest-box area. Aggressive pecking and severe feather pecking correlated positively. We assume that vigorous and painful AP were an additional stress factor, especially in non-beak-trimmed flocks, leading to more SFP in due course. Beak trimming had a reducing effect on SFP. However, our results showed that non-beak-trimmed flocks could be kept without major outbreaks of SFP.

## 1. Introduction

Aviary and free-range husbandry systems are considered to be more animal-friendly than cage systems because the animals can show a broader range of natural behaviors (e.g., dustbathing) [1,2]. Severe feather pecking (SFP) and cannibalism occur in all husbandry systems [3]. However, according to a survey among egg producers in Norway, ‘problematic behavior’, including feather pecking, was reported more frequently in aviary systems than in furnished cages [4]. Outbreaks of SFP or cannibalism (or both) are difficult to control [3] and indicate that the husbandry environment does not satisfy the needs of the animals living in it [2,5]. In a survey in Germany, 18.5% of farmers reported problems with feather pecking [6]. SFP is defined as pecking or pulling and sometimes eating the feather of another hen. The receiver usually shows agonistic behavior [7] because the pecking and pulling is painful [3,8]. SFP is not motivated by aggression and should not be confused with aggressive behavior [9,10,11]. According to the literature, there is no connection between the occurrence of SFP and agonistic behaviors such as resource or rank aggression [12,13]. The occurrence of SFP depends on various risk factors [14,15], but no definite etiology has been identified for this multifactorial problem [3,15,16]. Stress [3,17] and frustration [18] have an influence on the occurrence of SFP. An overview of the results of investigative and field studies suggests that 16 factors during the laying period are confirmed, including the provision of litter, of enough perches and access to a free range [19].

Larger flocks show higher SFP rates than smaller flocks [7,20]. A rising stocking density and flock size may lead to increasing problems [21]. However, in another study, the highest SFP rate was described in the smallest flock with the lowest stocking density [22]. The effect of stocking density and flock size during the laying period has not been analyzed thoroughly. Some authors found an influence of these factors [23,24]; others did not [25,26]. Although the provision of a litter area and a winter garden or free range may have a positive effect on reducing SFP [27], these areas must be attractively designed [28]. A study of 26 organic farms in the Netherlands indicated that SFP did not occur anymore if two thirds of the hens used the free range [29]. Therefore, a poor utilization of the free range (less than 50% of the hens) may be considered a risk factor for SFP [30]. The results of a survey of 107 flocks in eight European countries suggest that daily access to a free range and improved feeding may reduce the occurrence of SFP in organic laying hens [31]. Non-beak-trimmed Lohmann Brown, Lohmann Selected Leghorn and White Leghorn hens showed SFP mainly on perches, followed by the litter area and the aviary floors with feeders and drinking nipples [1,32]. On the other hand, the provision of perches gives subordinate hens the possibility to avoid dominant hens and reduces the frequency of agonistic interactions [33]. Another study found SFP predominantly in the litter area (63%) in comparison with the perches (37%), in 16 Hisex White flocks [7]. This finding supports the hypothesis that SFP can be interpreted as redirected litter pecking or foraging pecking [34,35]. The pecking patterns of different pecking activities (e.g., foraging or pecking against novel objects) differ significantly from each other, and SFP resembles only foraging and no other pecking activity [36]. SFP may occur if no adequate foraging stimulus is provided and conspecifics are used as substitutes [37]. Indeed, a negative correlation was found between litter pecking and SFP in connection with raised anxiety level in hens [38]. A meta-analysis confirmed the positive effect of environmental enrichment on the reduction of SFP [39]. The extent of plumage damage due to SFP also varies between hybrid lines [40,41]. Genetic variations are likely to be responsible for differences in the pecking behavior of different strains [15,42,43,44]. However, there is not just one gene controlling the multifactorial problem of feather pecking [45,46]. One study examined the reaction of Lohmann Selected Leghorn and Dekalb White hens to a restricted environment and detected different behavior patterns and SFP rates: Lohmann Selected Leghorn hens scratched less on the ground and had a higher SFP rate [42]. Another study found the highest SFP rates for ISA Brown, followed by Lohmann Brown, whereas White Leghorn hens pecked significantly less [40]. Lohmann Selected Leghorn also had higher SFP frequencies than Dekalb White hens [42]. In a study with non-beak-trimmed hens, SFP in Lohmann Brown Plus hens increased with age. In contrast, SFP remained exceptionally low in Lohmann Brown Dual hens throughout the laying period [47,48].

Beak trimming has been criticized widely in recent years as a painful procedure that adjusts the animal to its housing system instead of adjusting the housing system according to the behavioral needs of the animal. Beak trimming may lead to acute and chronic pain [8,49] and restricts normal foraging behavior, including feeding [50,51,52,53,54], depending on the method used [54]. The procedure may have a reducing effect on plumage damages [55] and the frequency of SFP [28,56]. Lambton et al. [28] observed a mean number of 0.032 and 0.017 feather pecking bouts per hen per minute in non-beak-trimmed and beak-trimmed hens, respectively. These occurrences correspond to the results of Staack et al. [56], who found that approximately twice as many non-beak-trimmed hens displayed plumage damages in comparison with beak-trimmed hens. Furthermore, compared with beak-trimmed flocks, non-beak-trimmed flocks showed a lower prevalence of hens with good plumage condition around 32 weeks of age [57]. In some EU countries, such as Germany and Austria, the majority of farmers stopped the beak trimming practice and have since kept non-beak-trimmed hens [58]. Several European countries are discussing a nationwide ban of beak trimming [57,59], and beak trimming is prohibited in Finland [60].

Surveys of commercial farms usually use the extent of plumage damage as an indicator of feather pecking [48]. Investigative studies with behavioral observations usually assess small units with a restricted number of animals [61]. Therefore, we conducted this study to gain insight into the actual pecking behavior of laying hens kept on commercial farms with flock sizes common in practice. We observed aggressive behavior and severe pecking behavior of non-beak-trimmed and beak-trimmed hens and investigated possible influencing factors on the occurrence of SFP.

## 2. Materials and Methods

### 2.1. Animals and Farms

The study took place on 8 commercial laying hen farms in Bavaria, Germany. Each farm had one flock of non-beak-trimmed laying hens (Flocks 1–8), and Farms 1 and 7 had an additional flock of beak-trimmed layers. The animals were kept in non-cage housing systems, partially with access to a winter garden or a free range or both (Table 1). The study was part of a larger project, which encompassed 16 farms in total, including the rearing farms. Each laying farm was visited 3 times during the laying period. During each visit, we recorded management, microclimate and animal health data [62,63]. 

We observed non-beak-trimmed flocks of the following hybrid lines: Lohmann Brown (LB) Classic, Lohmann Selected Leghorn (LSL) Classic, Bovans Brown (BB) and Dekalb White (DW). On 2 farms, we observed an additional flock of beak-trimmed hens of the same hybrid lines under identical management and housing conditions (Table 1). All animals were reared on conventional farms in aviary housing systems without access to a winter garden or free range before being transferred to the laying farm between the 15th and 25th week of life. During the laying period, the behavior of the animals was recorded in 3 observation periods (OPs): OP 1, at the peak of the laying period between the 28th and 33rd week of life; OP 2, in the middle of the laying period between the 42nd and 48th week of life; and OP 3, at the end of the laying period between the 63rd and 68th week of life (Table 1). All flocks were provided with litter (straw or wood shavings), and Flocks 1, 3, 4, 6 and 8 were provided with additional environmental enrichment (alfalfa or straw bales, pecking blocks, grains in the litter). All flocks were fed via automatic feeding lines and had drinking nipples. All farms used commercial laying-hen mesh feed, but consumption was only recorded for Flocks 1 and 2. Flocks 1 and 8 were not vaccinated during the laying period, and the vaccination status of Flock 4 was unknown. The other flocks were vaccinated against Newcastle Disease (NCD) and Avian Infectious Bronchitis (IB) as follows: Flock 2: NCD (5×) and IB (3×); Flock 3: NCD (1×) and IB (4×); Flock 5: NCD and IB (2× each); Flock 6: IB (2×); Flock 7: IB (1×).

### 2.2. Video Observation

For the video observation, we used VTC-E220IRP SANTEC color cameras with infrared and light-emitting diodes (Santronic AG, Wangen, Switzerland). The camaras automatically switched to infrared mode once the illuminance fell below 5 lx. Therefore, we were able to record both daytime and nighttime videos. Recording and video analysis were performed using the IP-Video and Alarm-Management software by IndigoVision and the associated hardware (IndigoVision encoder boxes and Ethernet Switch 8 Port, Indigo Vision Group Ltd., Milton Bridge, Scotland, UK).

We installed 6 to 8 cameras per flock, at least 2 cameras in each of the 3 functional areas: perches, nest-box area and litter area. Depending on the housing system and the availability of a winter garden, we used extra cameras to achieve a better supervision of the flock (Table 1). We distributed the cameras evenly across the stable and took care that the areas covered by each camera did not overlap each other. A detailed description of the layout of the farms, including camera position, can be found in Lenz [63].

The analyses encompassed two 24 h days within 2 weeks per camera and OP. We chose days without unusual disturbance, such as the provision of new litter. The nests closed automatically in the evenings. This is the reason why we could not install cameras inside the nest itself, but we observed the area in front of the nest boxes, including the entrance. The winter gardens were not continuously open; therefore, there were fewer hours to be analyzed. Altogether, we used 6520 scans and 476.7 h of video recordings (mean values per flock and OP: 217 scans and 15.9 h). 

An actively pecking hen is referred to as an “actor,” and a hen that is being pecked at, as a “receiver.” The ethogram (Table 2) describes the definition of the behavior of the actors and the reactions of the receivers. 

### 2.3. Video Analyses

For the video analysis, we used methods as described by Martin and Bateson [64]. A continuous recording was performed for the first 5 min of each hour during the light phase. The pecking behavior was recorded using behavior sampling. An interaction was assessed as finished if the actor stopped pecking and showed another behavior, moved on to another receiver or pecked at another body region of the same receiver. The interaction was also defined as finished if the receiver fled and was followed by the actor. All coding was done by one trained observer who was aware of the treatment of the hens.

### 2.4. Statistics

All cameras simultaneously filmed non-overlapping areas in the stable, so the same hen could not be seen on 2 cameras in the same OP. Therefore, each camera was rated as a statistical sample for the pecking activity of the hybrid line in question (non-beak-trimmed flocks: *n* = 82 per OP; beak-trimmed flocks: *n* = 18 per OP). For the calculation of correlations, we used a mean flock value per hybrid line and farm (non-beak-trimmed: *n* = 13; beak-trimmed: *n* = 3) or per hybrid line and the functional areas: perches, litter area and nest-box area (non-beak-trimmed: *n* = 39; beak-trimmed: *n* = 9).

We used LibreOffice Calc for the preparation of the raw data. The statistical analysis was performed with IBM SPSS Statistics 22 and 26.0. For independent samples in more than 2 categories (comparative analyses of hybrid lines and functional areas), we performed an analysis of variance according to Kruskal–Wallis with, multiple comparisons in pairs adjusted for multiple testing, according to Bonferroni. In the first step of this 2-staged test, we calculated whether there were significant differences between the categories in regard to the distribution of a variable. If significant differences were detected, the single categories were compared with each other and tested on significance in the second step. For comparisons of samples in 2 categories, we used the Mann–Whitney U-test. Differences during the laying period (OPs 1–3 summarized) were calculated by using Friedman 2-fold rank variance analyses for associated samples. In analogy to the Kruskal–Wallis procedure, this test was also 2-fold. For bivariate correlations, we used Spearman’s rho. Differences with a probability of error of *p* < 0.05 were marked significant. Additionally, we performed a multifactorial analysis using a generalized linear model for each functional area with SFP bouts per hen in 5 min as dependent variable and three independent variables: 1. availability of a free range or winter garden or both, 2. hybrid line, 3. stocking density (hens per square meter usable area). Since the dependent variable was not normally distributed, as confirmed by using the Shapiro-Wilk-test, we used the ordinal-logistic type of model for multinomial distributions. The three independent variables were tested pairwise for interactions.

## 3. Results

The following results refer to non-beak-trimmed hens only, except those in Section 3.4, which contains the results of a comparative study between beak-trimmed and non-beak-trimmed hens.

### 3.1. Occurrence and Influencing Factors of Severe Feather Pecking on Flock Level

**Figure 1:**
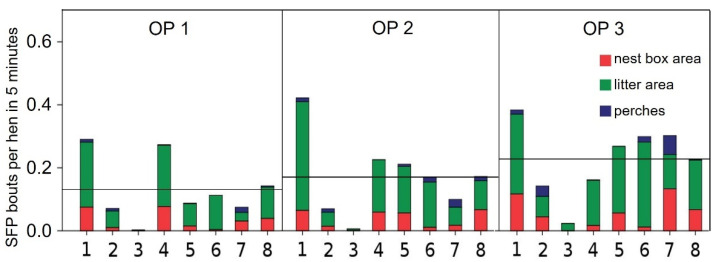
Mean rate of severe feather pecking (SFP) in non-beak-trimmed laying hens on flock level (Flocks 1–8). Observation Period (OP) 1: 28th to 33rd week of life; OP 2: 42nd to 48th week of life; OP 3: 63rd to 68th week of life. Horizontal black line = mean value.

All flocks showed SFP. The feather-pecking rate differed significantly (*p* < 0.001) in the pairwise comparison between all 8 flocks in all 3 functional areas (Figure 1). Flock 1 showed the highest feather-pecking rates throughout the study, whereas in Flock 3, SFP occurred very rarely. The mean rate of SFP increased during the laying period. Overall, we observed less SFP in all functional areas throughout the laying period in flocks with access to a free range or winter garden than in flocks housed in aviaries only (mean SFP rate per bird in 5 min of all flocks without access to a winter garden or a free range: 0.071; mean SPF rate per bird in 5 min of all flocks with access to a winter garden or a free range: 0.0543). This difference was found in all OPs (mean SFP rate per bird in 5 min of all flocks without access to a winter garden or a free range: perches: 0.018; litter area: 0.143; nest-box area: 0.0511; mean SPF rate per bird in 5 min of all flocks with access to a winter garden or a free range: perches: 0.005; litter area: 0.126; nest-box area: 0.032).The univariate analysis revealed a significant negative influence of the availability of a free range or winter garden or both on the feather-pecking rate on perches (*p* < 0.007) but not for the other functional areas (litter area: *p* = 0.781; nest-box area: *p* = 0.110). The multivariate analysis confirmed the results of the univariate analysis. Access to a winter garden or free range significantly reduced the SFP rate on perches (*p* = 0.001). This effect could not be shown for the litter area and the nest-box area. The stocking density (number of birds per usable square meter) had a significant influence on the SPF rate in the nest-box area (*p* = 0.001), The hybrid line had a significant effect on the SFP rate on perches and in the nest-box area (*p* = 0.001 each). Additionally, to the single effects of the stocking density and the hybrid line, there was a significant interaction between these two factors (*p* < 0.001). A high stocking density in combination with a hybrid line showing higher SFP rates resulted in a disproportionally high SFP rate.

We investigated several husbandry and management parameters that might have had an influence on the occurrence of SFP. First, we calculated correlations between the occurrence of SFP and the stocking densities. There were differences between the flocks in regard to the nest-box area (hens per square-meter nest box) and the available usable area per hen (hens per square meter of usable area). Therefore, we looked specifically into SFP rates in the litter area and the nest-box area because SFP was mainly observed in these areas. For this analysis, we used the mean values for SFP rates over the course of the laying period (OPs 1–3).

There was a positive correlation between the SFP rate and the number of hens per square meter of usable area. This correlation was significant regarding the pecking rates in the litter area (*rho* = 0.564; *p* = 0.045). The flock with the lowest stocking density (6.7 animals per square meter of usable area) showed the lowest feather-pecking rate (LB 0.010 feather pecking bouts per animal in 5 min), whereas the flocks with the highest stocking density (9.4 animals per square meter of usable area) had the highest feather-pecking rate in the litter area (DW 0.275 and BB 0.224 feather pecking bouts per animal in 5 min). We did not find a correlation between SFP and usable nest-box area. 

**Figure 2:**
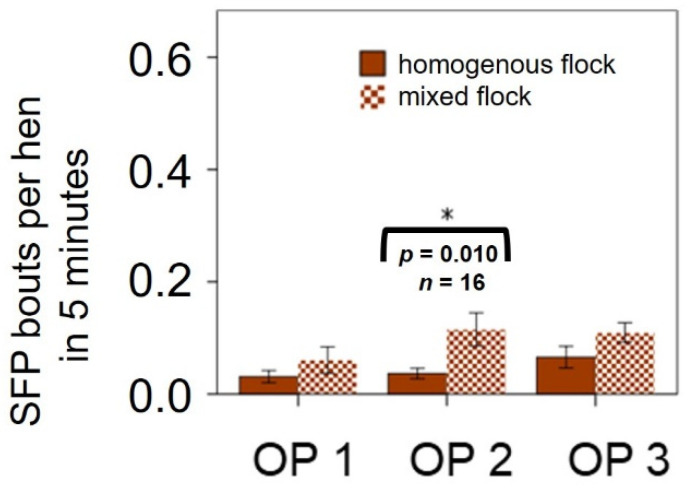
Severe feather pecking (SFP) rate in non-beak-trimmed Lohmann Brown (LB) hens in homogeneous (LB only) and mixed (LB + another hybrid line) flocks in the litter area during the laying period. Observation Period (OP) 1: 28th to 33rd week of life; OP 2: 42nd to 48th week of life; OP 3: 63rd to 68th week of life; *n* = number of cameras. Statistical test: Mann–Whitney U-test, significant: (*) = *p* ≤ 0.050. The bracket indicates a significant difference.

LB hens were kept both in homogeneous (LB only) and mixed (LB + another hybrid line) flocks. Therefore, we tested whether this hybrid line behaved differently, depending on how they were kept. We observed higher SFP rates in the mixed flocks, and the difference was significant in OP 2 (Figure 2).

### 3.2. Severe Feather Pecking in the Different Hybrid Lines and Functional Areas

**Figure 3:**
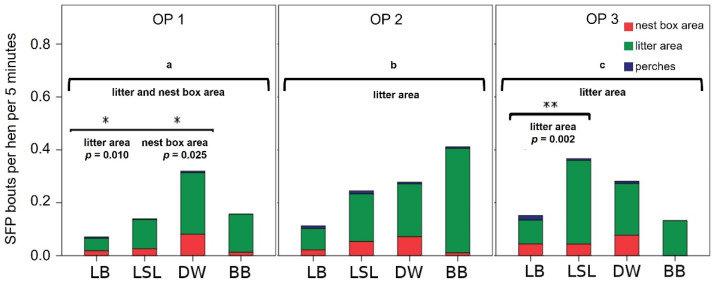
Mean occurrence of severe feather pecking (SFP) in non-beak-trimmed hybrid lines in different functional areas during three observation periods (OPs). Statistical test for differences between the hybrid lines: Kruskal–Wallis test, 1- or 2-fold (general and pairwise), significant: (*) = *p* ≤ 0.050; (**) = *p* < 0.010. General: comparison of the pecking behavior between the different laying hen hybrids in the functional areas in general; a = significant differences between the laying hen hybrids in the litter area and the nest-box area in OP 1; b and c = significant differences between the laying hen hybrids in the litter area in OP 2 and OP 3. Pairwise: comparison of laying-hen hybrids in pairs in functional areas with significant differences; bracket between the laying hen hybrids = significant *p*-value of the pairwise tests. OP 1: 28th to 33rd week of life; OP 2: 42nd to 48th week of life; OP 3: 63rd to 68th week of life. LB = Lohmann Brown (*n* = 45 cameras), LSL = Lohmann Selected Leghorn (*n* = 19 cameras), DW = Dekalb White (*n* = 12 cameras), BB = Bovans Brown (*n* = 6 cameras).

We observed SFP in all hybrid lines and all functional areas, with 2622 pecking bouts in total. Figure 3 shows the mean number of SFP bouts during the light phase. Overall, DW hens showed the highest feather-pecking rate, and LB hens, the lowest. In OP 2, BB hens had the highest rate, and in OP 3, LSL hens were pecking most frequently. Between 13% and 33% feather-pecking bouts were repeated bouts, depending on the hybrid line. LSL hens showed the highest percentage of repeated feather-pecking bouts. DW hens showed the most, and LB hens, the least feather pecking bouts.

Significant differences between the hybrid lines were found in the litter area and partially in the nest-box area. In OP 1, we found a significant difference between LB and DW hens in the litter area (*p* = 0.010) and in the nest-box area (*p* = 0.025). OP 3 revealed a significant difference between LB and LSL hens in the litter area (*p* = 0.010) (Figure 3). In the progress of the laying period, both LB and LSL hens showed a clear increase in the SFP rate. This increase was mainly due to significant increases in the feather-pecking rates in the litter area between OP 1 and OP 3 (LB: *p* = 0.008; LSL: *p* = 0.002). Furthermore, LB hens had an increasing SFP rate in the nest-box area and on perches. DW and BB hens did not show this development. They showed no significant differences during the laying period. 

All hybrid lines had peaks of SFP in the middle of the light phase between the 7th and 9th hour. The feather-pecking rates usually decreased in the afternoon, except for LB hens, which showed high feather-pecking rates towards the end of the light phase. All hybrid lines showed the highest feather-pecking rates in the litter area, followed by the nest-box area, with the lowest rates on perches. These differences were significant in all OPs together, with the exception of BB hens. The pairwise comparison of the functional areas between LB, LSL and DW hens resulted in the following significant differences between litter area and perches: LB: *p* < 0.001; LSL: *p* < 0.001; DW: *p* = 0.010 and between the litter area and the nest-box area: LB: *p* = 0.037 (Table 3).

### 3.3. Correlation of Aggressive Pecking and Severe Feather Pecking

We observed aggressive pecking (AP) in all flocks and all functional areas, with 3981 pecking bouts in total. The highest AP rates were found in the nest-box area, followed by the litter area and the perches. The difference between the functional areas was significant for all hybrid lines except BB (Table 4).

The pairwise comparison of the hybrid lines, LB, LSL and DW, revealed significant differences between the perches and the nest-box area (LB: *p* < 0.001; LSL: *p* = 0.007; DW: *p* = 0.043). For LB und LSL, the difference between the perches and the litter area was also significant (LB: *p* < 0.001; LSL: *p* = 0.010). Finally, we analyzed whether there was a correlation between the occurrence of AP and SFP. We found a significant positive correlation between AP and SFP in all OPs: OP 1: rho = 0.580, *p* < 0.001; OP 2: rho = 0.486, *p* = 0.002; and OP 3: rho = 0.482, *p* = 0.002 (*n* = 39).

### 3.4. Effect of Beak Trimming on the Occurrence of Aggressive Pecking and Severe Feather Pecking

We compared the pecking behavior of the hens on two farms that kept a flock of beak-trimmed and non-beak-trimmed hens hatched at the same time and raised under the same husbandry and management conditions. Both LB and DW hens showed, by tendency, more AP in the non-beak-trimmed than in the beak-trimmed flocks. BB hens had a tendency for a higher AP rate in the beak-trimmed than in the non-beak-trimmed flock on perches and in the nest-box area. As differences were not statistically significant, they need to be interpreted carefully.

**Figure 4:**
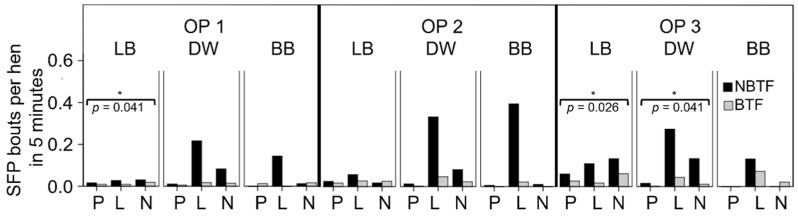
Occurrence of severe feather pecking (SFP) in beak-trimmed flocks (BTF) and non-beak-trimmed flocks (NBTF) in the functional areas, perches (P), litter area (L) and nest-box area (N), assessed in Observation Periods (OPs) 1–3. Statistical test: Mann–Whitney U-test, significant: (*) = *p* ≤ 0.050. The brackets indicate significant differences between BTF and NBTF. OP 1: 28th to 33rd week of life, OP 2: 42nd to 48th week of life, OP 3: 63rd to 68th week of life. LB = Lohmann Brown, DW = Dekalb White, BB = Bovans Brown. *n* = 12 cameras per OP and hybrid line.

Non-beak-trimmed LB and DW hens had, especially in the litter area, a higher SFP rate than their beak-trimmed counterparts. LB and DW hens showed the same pattern in other functional areas. Beak-trimmed BB hens showed higher SFP rates than non-beak-trimmed BB hens but on a very low level. The difference between non-beak-trimmed and beak-trimmed hens was significant for LB in OP 1 (*p* = 0.041) and OP 3 (*p* = 0.026) and for DW in OP 3 (*p* = 0.041). In beak-trimmed flocks, SFP in the litter area was reduced by more than half in comparison with non-beak-trimmed flocks (Figure 4).

## 4. Discussion

SFP was observed in all flocks and in all hybrid lines (including beak-trimmed flocks) similarly to other studies and reviews [3,31,56]. We found differences in extent, localization and development over time.

There was a significant positive correlation between the feather-pecking rate and a rising stocking density (usable area), especially in the litter area. After calculating the actual stocking density per flock, we found a range from 6.7 to 9.4 hens per square meter of usable area. This finding was not expected because all farms kept their animals according to the German Animal Welfare Act, which specifies a maximum stocking density of 9 hens per usable square meter [65]. Flock 1 had the highest SFP rates and was the only flock exceeding the maximum stocking density of 9 hens per square meter, and Flock 3, in contrast, had both the lowest SFP rate and the lowest stocking density. We assume that the stocking density was an influencing factor for SFP in this study, as has been described previously [21]. The provision of a free range or a winter garden seemed to have a positive effect on the prevention of SFP because flocks without a free range or winter garden showed an higher overall pecking rate. The difference was significant on perches. A reason for this finding could be that the winter gardens and free ranges were an additional space and not used for calculating the stocking density. The provision of this additional space reduced the stocking density during the day when the birds were active. The results of many previous surveys have pointed out that SFP could be reduced if are hens were motivated to show more foraging behavior [17,19,29,30]. However, this effect could not be seen in all flocks and OPs in our study, as Flocks 4, 5 and 6 had an above-average pecking rate despite provision of a free range or winter garden. This finding can probably be explained by differences in the utilization of the free range or winter garden, which was highest in Flock 3 (48%). Not the free range itself but a high usage is important to prevent SFP [28,29,30]. We did not find a correlation between the number of hens per square meter nest box and SFP in the nest-box area, although this parameter had an unexpectedly wide range (79–117 hens per square-meter nest box). However, we could not properly observe the hens in the nest—only in front of the nest entrance—owing to technical restrictions (nests closed automatically in the afternoon). A special characteristic of certain marketing strategies in laying-hen husbandry is to keep white and brown layers together in one flock. We found a significantly lower SFP rate in flocks with LB hens only (homogeneous flocks), especially in the litter area, compared with mixed flocks in which LB hens were housed together with hens of a white layer line. This result might be due to genetic differences in the general temperament and behavior of different hybrid lines [45,46,66]. Our findings are in line with the results of another study, which assumed a higher SFP rate of brown layers (Rhode Island Red) against the white layers (White Leghorn) in mixed flocks [67]. Some authors even suggest using different aviary designs for different hybrid lines [68]. Another study of similar Bavarian farms could not reproduce these findings [69]. More research is needed to verify whether keeping laying hens in mixed flocks might be a risk factor for SFP. Based on our results and the genetic differences between white and brown layers, we suggest not to keep laying hens in mixed flocks. If white and brown eggs need to be packed in one carton, the eggs of two separate flocks of white and brown layers could be mixed after collecting them. Other management factors should be looked into further in future studies, e.g., the influence of different lighting systems or the age of the birds when being transferred from the rearing facility to the laying barn.

Not surprisingly, we found differences in the pecking behavior between the different hybrid lines. Hybrid line had a significant effect on the pecking rate in the functional areas, “perches” and “nest-box area”. This finding is in line with field surveys [29,31] and experimental studies that aimed to determine a heritability of SFP [66,70,71]. According to one study, LB hens showed higher pecking rates than White Leghorn hens [40], an observation we could not confirm. In our study, LB hens had the lowest pecking rates (mean value: 0.112 pecking bouts per animal in 5 min) in comparison with the other hybrid lines. Another study found a higher SFP rate in LSL compared with DW hens. Our results showed an overall higher pecking rate in DW hens. This might be due to the genetic advancement in laying-hen breeding [45,46]. On the other hand, our sample size is too small to draw definite conclusions about differences in feather pecking behavior of different hybrid lines. We only observed white layers in mixed flocks, and we assume that this husbandry method has an influence on the animal behavior, too. Therefore, we cannot determine whether the hybrid line, the mixed flock or both was responsible for our results. We observed SFP, especially in the litter area. This finding emphasizes the importance of enough litter and regular litter renewal for the prevention of SFP [31]. Environmental enrichment in the litter area, such as straw bales or pecking blocks, may have an additional positive effect [39,72]. We found a correlation between SFP and AP throughout the laying period. This does not correspond with results of other studies that did not find an association between SFP and AP [12,13]. The underlying motivation for both behaviors is different [9,73]. Our video analyses revealed that AP was shown as reaction by the receiver of SFP bouts but never by the initiator of an interaction. We assume that AP is a common reaction to the painful plucking on the feather of the receiver. A high SFP rate may therefore lead to more agonistic behavior and may increase the overall stress level even further, which may trigger more SFP [17]. It is uncertain whether a high level of agonistic behavior leads to more SFP or if it is an indication of the occurrence of SFP. Flock 3, for example, had a high AP but a very low SFP rate. From our data, we could not calculate the percentage of defensive aggressive behavior of the total amount of AP. This would be an interesting research topic for further studies. Our study was conducted on eight commercial farms only; all data obtained should therefore be interpreted carefully. However, we believe that observations on commercial farms add valuable information toward the understanding and prevention of SFP.

Pecking behavior (both AP and SFP) differed between beak-trimmed and non-beak-trimmed birds. In both cases, non-beak-trimmed hens had a higher pecking rate, except for a higher AP rate in beak-trimmed than in non-beak-trimmed BB hens. Non-beak-trimmed hens showed more SFP than their beak-trimmed counterparts, especially in the litter area. There were also hints that there was a high SFP rate on the aviary levels, although we did not analyze this aspect in detail in this study. Further studies should investigate the animal behavior on the aviary levels in more detail. In the litter area, SFP in beak-trimmed hens was less than half as much as in non-beak-trimmed birds. This finding is coherent with a study that found that feather-pecking rates were approximately halved after beak trimming (from 0.032 down to 0.017 bouts per animal per minute) [28]. Other authors reported mixed results. Some flocks showed a similar reduction, whereas other flocks showed no difference between beak-trimmed and non-beak-trimmed animals [41,55]. We found the biggest difference in pecking behavior between beak-trimmed and non-beak-trimmed flocks in the litter area. Therefore, AP and SFP might influence each other in this functional area. Intact beaks probably lead to more plumage damage and pain. Different authors [17,18,21] have provided evidence that stress and frustration have a negative influence on SFP and cannibalism. We assume that vigorous and painful AP were an additional stress factor, especially in non-beak-trimmed flocks, leading to more SFP in due course. This could lead to a vicious cycle, with AP and SFP reinforcing each other over time. Beak trimming had a reducing effect on the pecking behavior in this study. Nonetheless, the overall lowest pecking rate of all flocks was found in a non-beak-trimmed flock. Non-beak-trimmed hens may be kept without major outbreaks of SFP if their housing and management conditions are adequate. In recent years, many influencing factors of this multifactorial problem have been identified [3,4,27,29,31] and incorporated into husbandry guidelines [19,24,74]. The experiences in some European countries, such as Austria and Germany, show that SFP in non-beak-trimmed flocks can be prevented if housing and management are adequate [62,69,75].

## 5. Conclusions

In conclusion, we observed severe feather pecking in all flocks and hybrid lines, both beak-trimmed and non-beak-trimmed. However, the frequency and development over time was very variable. Access to a winter garden or free range significantly reduced the SFP rate on perches (*p* = 0.001). The stocking density (number of birds per usable square meter) had a significant influence on the SPF rate in the nest-box area (*p* = 0.001), The hybrid line had a significant effect on the SFP rate on perches and in the nest-box area (*p* = 0.001 each). Our data suggest a positive influence of the stocking density (hens per usable square meter) and the provision of a winter garden or free range on the behavior of the birds (less feather pecking). In our study, hens in mixed flocks showed significantly more SFP than hens kept in homogeneous flocks, and LB hens had the lowest overall SFP rates compared with white layer lines. However, both the results on differences in the pecking behavior of the different hybrid lines and mixed flocks versus homogeneous flocks should be interpreted carefully, as we observed white layers in mixed flocks only. Non-beak-trimmed hens showed more SFP than their beak-trimmed counterparts, especially in the litter area. Aggressive pecking and severe feather pecking correlated positively. We assume that vigorous and painful AP were an additional stress factor, especially in non-beak-trimmed flocks, leading to more SFP in due course. This could lead to a vicious cycle, with AP and SFP reinforcing each other over time. Our results emphasize the importance of the provision of enough space, especially in the litter area, including suitable foraging material. An additional winter garden or free range that is extensively used by the hens seems to be beneficial for the animal behavior. Beak trimming had a reducing effect on SFP. However, our results showed that non-beak-trimmed flocks could be kept without major outbreaks of SFP. 

## Figures and Tables

**Table 1:** Overview of the flocks, their housing conditions and the distribution of the video cameras. All flocks were housed in aviaries.

Flock	Additional Space	Hatch Date (DD.MM.YYYY)	OP ^1^: Week of Life	Hybrid Line(s)	Flock Size NBT(BT)	Number of Hens Per Square Meter Usable Area	Number of Hens Per Square Meter Nest	Camera Positions	Daylight/Artificial Light Type/Color
1	n.a.	26.03.2012	OP 1: 29–31 OP 2: 44–46 OP 3: 64–66	DW (85%) BB (15%)	3589 (3665)	9.4	108	2× perch, 2× litter area, 2× nest	No/HFFLT/white
2	n.a.	06.10.2011	OP 1: 29–31 OP 2: 46–48 OP 3: 64–68	LB	4250	8.5	92	3× perch, 3× litter area, 2× nest	Yes/HFFLT, LED/white
3	Free range + winter garden	27.01.2012	OP 1: 29–31 OP 2: 43–45 OP 3: 64–66	LB	4212	6.7	117	2× perch, 2× litter area, 2× nest, 1× winter garden	Yes/HFFLT, lightbulb, LED/white
4	Free range + winter garden	04.05.2012	OP 1: 31–33 OP 2: 42–44 OP 3: 63–65	LB (50%) DW (50%)	1450	8.9	79	2× perch, 2× litter area, 2× nest, 1× winter garden	Yes/LED/white
5	Free range	20.02.2012	OP 1: 30–32 OP 2: 46–48 OP 3: 64–66	LB (50%) LSL (50%)	2004	8.5	116	2× perch, 3× litter area, 2× nest	Yes/lightbulbs/yellow
6	Free range + winter garden	31.12.2011	OP 1: 28–30 OP 2: 44–46 OP 3: 64–68	LB (75%) LSL (25%)	2000	7.5	104	2× perch, 2× litter area, 2× nest, 1× winter garden	Yes/HFFLT, lightbulbs/white
7	n.a.	31.12.2011	OP 1: 29–31 OP 2: 44–46 OP 3: 63–65	LB	4500 (5000)	7.8	98	2× perch, 2× litter area, 2× nest	No/HFFLT, lightbulbs/white, yellow
8	winter garden	14.01.2012	OP 1: 29–31 OP 2: 44–46 OP 3: 64–66	LB (50%) LSL (50%)	1481	8.6	110	2× perch, 2× litter area, 2× nest, 1× winter garden	Yes/HFFLT, lightbulbs/white

^1^ Abbreviations: DW = Dekalb White, BB = Bovans Brown, LB = Lohmann Brown, LSL = Lohmann Selected Leghorn, NBT = non-beak-trimmed, BT = beak-trimmed (2 farms only), HFFLT = high-frequency fluorescent light tube, LED = light-emitting diode, OP = observation period.

**Table 2:** Ethogram used for the video analyses, with definitions of the pecking behavior shown by the actor (pecking hen).

Behavior of the Actor	Definition
Aggressive pecking	Forceful pecking, normally upward-down directed towards the head. Associated with a strong reaction of the receiver (flight, flirt or fight).
Severe feather pecking	Violent pecking directed towards the plumage or bald skin areas. Visible plucking of one or more feathers and/or avoiding or aggressive reaction of the receiver.

**Table 3:** Severe feather pecking in the three functional areas, perches (P), litter area (L) and nest-box area (N), shown for Observation Periods (OPs) 1–3 and for the whole laying period (mean value for OPs 1–3) ^1^.

Layer Line	Comparison	OP 1	OP 2	OP 3	Mean Value
LB (*n* = 45)	General	*p* = 0.006 **	*p* < 0.001 ***	*p* = 0.001 ***	*p* < 0.001 ***
	Pairwise	P < L ** (*p* = 0.004) P < N (n.s.) L > N (n.s.)	P < L *** (*p* = 0.001) P < N (n.s.) L > N ** (*p* = 0.009)	P < L *** (*p* < 0.001) P < N (n.s.) L > N (n.s.)	P < L *** (*p* < 0.001) P < N (n.s.) L > N * (*p* = 0.037)
LSL (*n* = 19)	General	*p* = 0.003 **	*p* = 0.002 **	*p* = 0.001 **	*p* = 0.001 **
	Pairwise	P < L ** (*p* = 0.002) P < N (n.s.) L > N (n.s.)	P < L ** (*p* = 0.002) P < N (n.s.) L > N (n.s.)	P < L *** (*p* = 0.001) P < N (n.s.) L > N (n.s.)	P < L *** (*p* < 0.001) P < N (n.s.) L > N (n.s.)
DW (*n* = 12)	General	*p* = 0.007 **	*p* = 0.094 (n.s.)	*p* = 0.055 (n.s.)	*p* = 0.012 *
	Pairwise	P < L ** (*p* = 0.005) P < N (n.s.) L > N (n.s.)	Not applicable	Not applicable	P < L * (*p* = 0.010) P < N (n.s.) L > N (n.s.)
BB (*n* = 6)	General	*p* = 0.123 (n.s.)	*p* = 0.165 (n.s.)	*p* = 0.091 (n.s.)	*p* = 0.165 (n.s.)

^1^ Statistical test: Kruskal–Wallis test, 2-fold (general and pairwise); n.s. = not significant (*p* > 0.050); * = significant (*p* ≤ 0.050); ** = significant (*p* ≤ 0.010); *** = significant (*p* ≤ 0.001). General: Calculation of significant differences between the functional areas in general. Pairwise: Pairwise calculation within the groups with significant differences; > and < = higher and lower feather-pecking rate, respectively. Timing of observation: OP 1 = 28th to 33rd week of life, OP 2 = 42nd to 48th week of life, OP 3 = 63rd to 68th week of life. Layer lines: LB = Lohmann Brown, LSL = Lohmann Selected Leghorn, DW = Dekalb White, BB = Bovans Brown, *n* = number of cameras.

**Table 4:** Aggressive pecking in the three functional areas, perches (P), litter area (L) and nest-box area (N), shown for Observation Periods (OPs) 1–3 and for the whole laying period (mean value for OPs 1–3) ^1^.

Layer Line	Comparison	OP 1	OP 2	OP 3	Mean Value
LB (*n* = 45)	General	*p* < 0.001 ***	*p* < 0.001 ***	*p* < 0.001 ***	*p* < 0.001 ***
	Pairwise	P < L *** (*p* < 0.001) P < N *** (*p* < 0.001) L > N (n.s.)	P < L *** (*p* < 0.001) P < N *** (*p* < 0.001) L < N (n.s.)	P < L *** (*p* < 0.001) P < N *** (*p* < 0.001) L < N (n.s.)	P < L *** (*p* < 0.001) P < N *** (*p* < 0.001) L < N (n.s.)
LSL (*n* = 19)	General	*p* = 0.003 **	*p* = 0.001 ***	*p* = 0.003 **	*p* = 0.003 **
	Pairwise	P < L * (*p* = 0.010) P < N ** (*p* = 0.007) L < N (n.s.)	P < L * (*p* = 0.038) P < N *** (*p* = 0.001) L < N (n.s.)	P < L ** (*p* = 0.006) P < N * (*p* = 0.012) L < N (n.s.)	P < L ** (*p* = 0.005) P < N * (*p* = 0.014) L < N (n.s.)
DW (*n* = 12)	General	*p* = 0.024 *	*p* = 0.023 *	*p* = 0.024 *	*p* = 0.018 *
	Pairwise	P < L (n.s.) P < N * (*p* = 0.043) L > N (n.s.)	P < L (n.s.) P < N * (*p* = 0.032) L > N (n.s.)	P < L (n.s.) P < N * (*p* = 0.043) L > N (n.s.)	P < L (n.s.) P < N * (*p* = 0.018) L > N (n.s.)
BB (*n* = 6)	General	*p* = 0.156 (n.s.)	*p* = 0.123 (n.s.)	*p* = 0.095 (n.s.)	*p* = 0.180 (n.s.)

^1^ Statistical test: Kruskal–Wallis test, 2-fold (general and pairwise), n.s. = not significant (*p* > 0.050); * = significant (*p* ≤ 0.050); ** = significant (*p* < 0.010); *** = significant (*p* ≤ 0.001). General: Calculation of significant differences between the functional areas in general. Pairwise: Pairwise calculation within the groups with significant differences: > and < = higher and lower feather-pecking rate, respectively. Timing of observation: OP 1 = 28th to 33rd week of life, OP 2 = 42nd to 48th week of life, OP 3 = 63rd to 68th week of life. Layer lines: LB = Lohmann Brown, LSL = Lohmann Selected Leghorn, DW = Dekalb White, BB = Bovans Brown, *n* = number of cameras.

## Data Availability

The data presented in this study are available on request from the corresponding author. The data are not publicly available due to legal resons.
